# Supplementary material for: The Construction and Exploration of a Comprehensive MicroRNA Centered Regulatory Network in Foxtail Millet (Setaria italica L.)
Source: Front Plant Sci. 2022 May 6;13:848474. doi: 10.3389/fpls.2022.848474 (PMC9121102; doi:10.3389/fpls.2022.848474)
Supplement: Supplementary file 4 [file Data_Sheet_4.zip › Sit-miRN3023b_Seita.2G265400.1_812_TPlot.pdf]

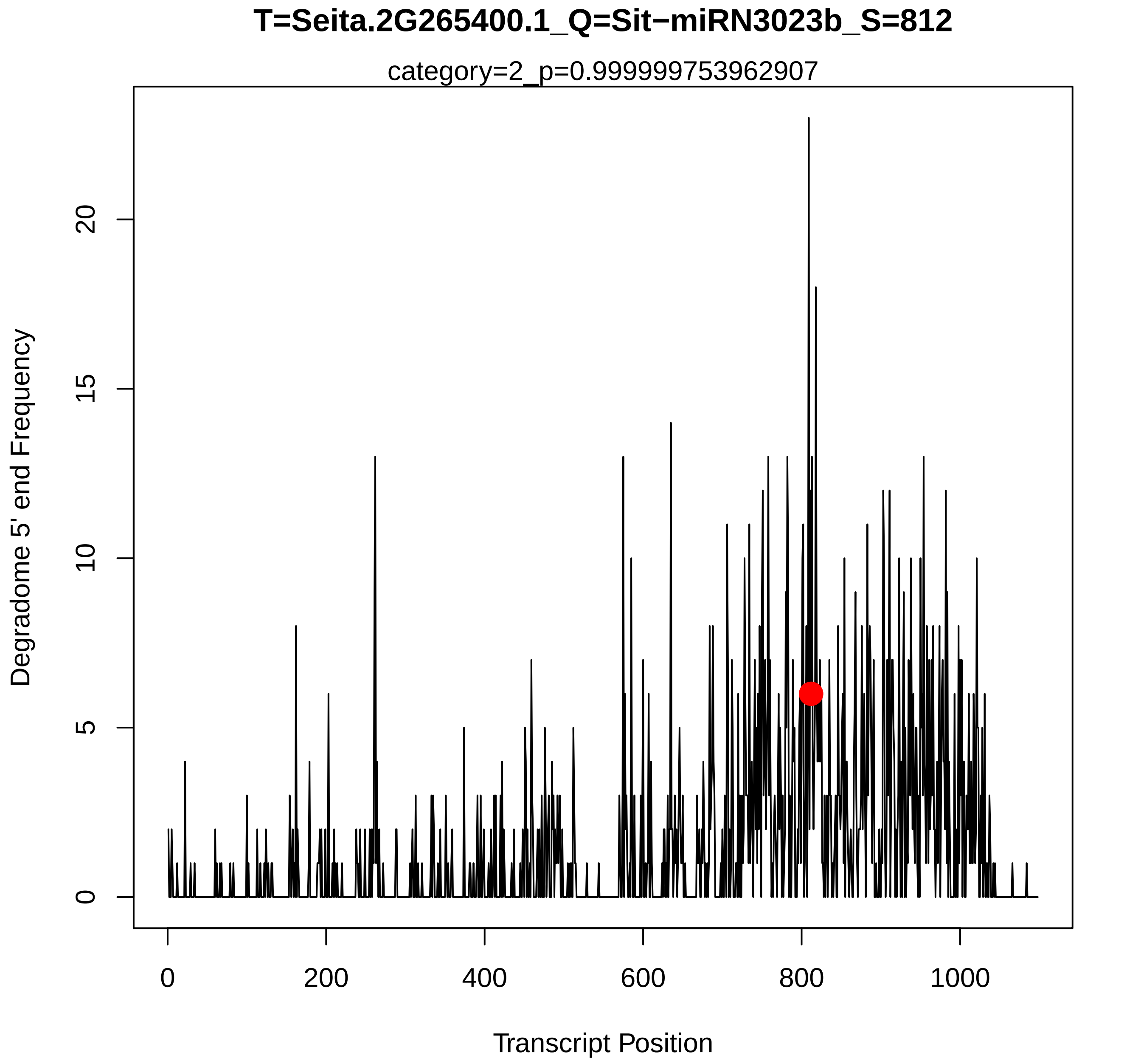
T=Seita.2G265400.1_Q=Sit−miRN3023b_S=812
category=2_p=0.999999753962907
Degradome 5' end Frequency
Transcript Position
0
5
10
15
20
0
200
400
600
800
1000
